# Dataset on travellers’ acceptance of border control technologies: Insights from METICOS pilot trials

**DOI:** 10.1016/j.dib.2025.111278

**Published:** 2025-01-08

**Authors:** Sarang Shaikh, Sule Yildirim Yayilgan, Erjon Zoto, Mohamed Abomhara

**Affiliations:** Department of Information Security and Communication Technology, Norwegian University of Science and Technology (NTNU), 2815 Gjøvik, Norway

**Keywords:** Questionnaire, Technology acceptance, Border control technology, User perceptions, Survey, Artificial intelligence

## Abstract

This paper presents a dataset from the METICOS^1^ project pilot trials related to the acceptance of border control technologies. There was total five pilots held at Tallin Airport, Athens International Airport, Larnaca International Airport, Border Crossing Point Moravita Land Border, and Vienna International Airport. The dataset consists of the data collected using an online questionnaire (survey) to assess travellers’ technology acceptance in terms of their demographics, profiles, and user perceptions along with operational information of border control technologies during their use. The questionnaire items together with their responses are paired samples collected after the travellers use the technology at the METICOS project pilot trials held in five different locations (i.e. four airports, and one land border) during the period of January to August 2023. The ABC-gates were used to assess technology acceptance during all the pilot trials provided by pilot partner of the METICOS. The total size of the dataset is 147 instances and is well-suited for quantitative analysis of assessing technology acceptance indicators for border control technologies. This information can aid policymakers and border control authorities in enhancing the acceptance and the use of these technologies at various border crossing points across Europe.

Specifications Table

**Table:** 

Subject	Computer Science
Specific subject area	Artificial Intelligence
Type of data	*.*xlsx file (filtered dataset from questionnaire – removed occupation, and technology name)
Data collection	The survey was conducted using an online questionnaire created with the Flask framework in Python and hosted on the METICOS server. It included stepwise questions covering traveller demographics, profiles, perceptions, operational details, and their technology acceptance score. The questionnaire design was informed by prior research, expert opinions, and METICOS end-user partners. Data was collected during METICOS pilot trials from January to August 2023, with 147 participants. The questionnaire is available in the data repository.
Data source location	METICOS pilot trials 1. Estonia^2^ → Tallin Airport (23.01.2023) 2. Greece^3^→ Athens International Airport (27.04.2023) 3. Cyprus^4^→ Larnaca International Airport (09.05.2023) 4. Romania^5^ → Border Crossing Point Moravita Land Border (30.05.2023) 5. Austria^6^ → Vienna International Airport (17.08.2023)
Data accessibility	Repository name: EU Open Research Repository (Pilot) supported by Zenodo Data identification number: 10.5281/zenodo.12759705 Direct URL to data: https://zenodo.org/records/12759705
Related research article	N*/A*

## 1 Value of the Data

• To the best of our knowledge, this dataset is the first of its kind addressing technology acceptance of border control technologies in a European context. It fills a significant gap in existing research and can serve as a benchmark for future studies in this area.
• The dataset contains traveller technology acceptance scores from the METICOS pilot trials regarding the border control technologies acceptance which are beneficial for understanding public perceptions, attitudes, and technology acceptance of adults (18 years and over) towards border control technologies to ensure user acceptance, secure positive societal impact (smooth travel experience) and maximize border control process efficiency.
• The data can be used in quantitative research to analyze the data for understanding factors influencing the technology acceptance of border control technology and for developing algorithms for predicting technology acceptance.
• Researchers across various fields such as social science, policy making, computer science, engineering and design and public policy can utilize the dataset to explore the intersection of technology, user experience, and societal impact in the context of border control.
• The data can support research into policymaking for improving the use of border control technologies, enabling policymakers to design strategies that align public perceptions with the overall technology acceptance.
• The policymakers at the border crossing points can leverage this data to design public awareness campaigns or training programs aimed at improving acceptance of border control technologies.
• The data can further provide meaningful input to machine learning algorithms to predict technology acceptance and identify key predictive input features influencing technology acceptance which is one of the promising analytical approaches.

## 2 Background

The EU-funded METICOS project aims to create an up-to-date technology acceptance classification scheme as well as a societal and ethical impact dashboard of border control technologies, to empower three major sub-divisions of the wider border control sector: travellers, border control authorities and service providers. The project duration was from September 2020 to October 2023 comprising of participant organizations involving 14 research, public, and private sectors from 8 different European countries (Cyprus, Greece, Norway, Belgium, Romania, Austria, Italy, and Estonia). Overall, the major goal of METICOS project is to create a holistic solution to address challenges for border management, both as regards societal, technological acceptance and efficiency. This dataset addresses the technology acceptance classification part of the METICOS project. To this date, to the best of our knowledge, there exists no such dataset except the works done as a part of METICOS project addressing the technology acceptance of border control technologies in any context (i.e. social, cultural, ethical, etc.). The collection of this dataset using an online questionnaire was guided by five end-user partners (four airports and one land border) official partner of the METICOS project consortium. All of the data variables as a part of this dataset were carefully reviewed by the representatives of all the consortium partners to confirm their relevance/impact on technology acceptance of border control technologies.

## 3 Data Description

This section describes the dataset consisting of responses from volunteer travellers who participated into METICOS pilot trials for assessing the acceptance of border control technology. The dataset consists of responses from 147 travellers, representing a variable distribution of different demographics, profile attributes such as gender, age, nationality, education level, etc. Table 1 shows the list of all the data attributes collected into this dataset together with their short descriptions.

**Table 1:** Questionnaire data column labels,descirptions and modality (N = 22).

Label	Description	Modality
Gender	Gender of traveller	Categorical (Male; Female; Other)
Age	Age of traveller	Categorical (18–24; 25–34; 35–49; 50–64; 65+)
Nationality	Nationality of traveller	Categorical (All countries)
Education level	Education level of traveller	Categorical (No education; Primary; Secondary; Bachelors; Masters; Doctorate)
Familiarity with technology	Familiarity of traveller with border control technologies	Ordinal (No familiarity; Less familiar; Very familiar)
Travel frequency	Travel frequency of traveller in last two years	Ordinal (No previous travel; 1–9 travels; 10–49 travels)
Travel experience with technology	Travel experience of traveller using border control technologies	Ordinal (No experience; Little experience; Full experience)
Overall time to cross the border	Overall time the traveller took to cross the border using border control technology	Continuous (scale: seconds)
Waiting time	Waiting time of traveller in queue while reaching to the border control technology placement location	Continuous (scale: seconds)
Average people in the queue	Average number of travellers in queue while reaching to border control technology placement location	Continuous
Number of successful attempts	Number of successful attempts by traveller while using the border control technology	Continuous
Number of unsuccessful attempts	Number of unsuccessful attempts by traveller while using the border control technology	Continuous
Response time	Response or turn-around time of border control technology to make necessary decision	Continuous (scale: seconds)
Speed (Was the technology fast and quick when you tried to use it?)	Rating by the traveller for “speed” aspect of the border control technology	Ordinal (scale: Likert-scale from 1 to 5, where 1 = lowest rating and 5 = highest rating)
Interface (Was the interface of the technology easy to understand in terms of instructions and steps?)	Rating by the traveller for “interface” aspect of the border control technology	Ordinal (scale: Likert-scale from 1 to 5, where 1 = lowest rating and 5 = highest rating)
Malfunction (Was there any errors/complaints while using the technology?)	Rating by the traveller for “malfunction” aspect of the border control technology	Ordinal (scale: Likert-scale from 1 to 5, where 1 = lowest rating and 5 = highest rating)
User Interface (UI) Design (Was the user interface design of the technology comfortable?)	Rating by the traveller for “UI design” aspect of the border control technology	Ordinal (scale: Likert-scale from 1 to 5, where 1 = lowest rating and 5 = highest rating)
General	Rating by the traveller for “general” aspect of the border control technology	Ordinal (scale: Likert-scale from 1 to 5, where 1 = lowest rating and 5 = highest rating)
Sentiment	Sentiment of the traveller after using the border control technology	Categorical (positive; negative; neutral)
Emotion	Emotion of the traveller after using the border control technology	Categorical ((joy; surprise; anger; fear; sad; disgust))
Open feedback	Feedback from the traveller regarding their experience using the border control technology	Open Text
Technology acceptance score	Rating by the traveller for “technology acceptance” of border control technology based on their experience	Ordinal (Likert-scale from 1 to 5, where 1 = lowest technology acceptance and 5 = highest technology acceptance)

Table 2 shows data on the demographics and profile characteristics of travellers (i.e. participants) distributed into each demographic/profile type with each of their possible option values together with their ratio in the dataset.

**Table 2:** Travellers’ demographics and profiles characteristics (*n* = 147).

No	Category	Sub-category	Frequency	%
				
1	**Gender**	female	46	31.29 %
		male	94	63.95 %
		other	7	4.76 %
2	**Age**	18–24	48	32.65 %
		25–34	37	25.17 %
		35–49	45	30.61 %
		50–64	8	5.44 %
		65+	9	6.12 %
3	**Education Level**	bachelors	36	24.49 %
		doctorate	5	3.40 %
		masters	14	9.52 %
		no education	26	17.69 %
		primary	14	9.52 %
		secondary	52	35.37 %
4	**Familiarity with technology**	less familiar	66	44.90 %
		no familiarity	33	22.45 %
		very familiar	48	32.65 %
5	**Travel frequency**	10–49 travel	33	22.45 %
		1–9 travel	77	52.38 %
		no previous travel	37	25.17 %
6	**Travel experience with technology**	full experience	31	22.30 %
		little experience	62	44.60 %
		No experience	46	33.09 %

This dataset also includes operational/technological attributes collected for each participant such as “average people in the queue, overall time to cross the border, response time, number of successful attempts, waiting time, and number of unsuccessful attempts” while using the border control technology at METICOS pilot trials. Table 3 provides details on the summary statistics for these attributes.

**Table 3:** Operational and technological data attributes summary statistics (*n* = 147).

	Average people in the queue	Overall time to cross the border	Response time	Number of successful attempts	Waiting time	Number of unsuccessful attempts
count	147	147	147	147	147	147
mean	7.20	94.03	50.95	1.88	16.88	0.88
std	9.75	102.70	54.14	1.52	17.98	1.22
min	0	0	0	0	0	0
25 %	0	20	5	1	1	0
50 %	2	60	30	1	10	0
75 %	10	120	90	3	30	1
max	48	600	240	8	60	5

Finally, the dataset also shows the information from the participants in terms of their feedback and technology acceptance score for the border control technology being used during the METICOS pilot trials. The participants were asked to provide feedback about their experience for using the technology in different ways: 1) Rating on different aspects (i.e. speed, UI design, malfunction, interface, and general) of border control technology on a Likert-scale from 1 to 5; with step count of 1. 2) Sentiment after using the technology, 3) Emotion after using the technology, 4) Open text feedback about their experience in using the technology, and 5) Rating on overall technology acceptance score of border control technology on a Likert-scale from 1 to 5; with step count of 1. Table 4 shows some of the details about these attributes except the “open text feedback”.

**Table 4:** Travellers’ feedback, technology acceptance score statistics (*n* = 147).

Category	Sub-category	Rating	Frequency	%
Aspect	Speed	1	17	11.56 %
		2	8	5.44 %
		3	19	12.93 %
		4	35	23.81 %
		5	68	46.26 %
	UI Design	1	44	29.93 %
		2	11	7.48 %
		3	21	14.29 %
		4	38	25.85 %
		5	33	22.45 %
	Malfunction	1	72	48.98 %
		2	23	15.65 %
		3	11	7.48 %
		4	18	12.24 %
		5	23	15.65 %
	Interface	1	43	29.25 %
		2	8	5.44 %
		3	22	14.97 %
		4	27	18.37 %
		5	47	31.97 %
	General	1	41	27.89 %
		2	11	7.48 %
		3	16	10.88 %
		4	39	26.53 %
		5	40	27.21 %
Sentiment	negative		17	11.56 %
	neutral		9	6.12 %
	positive		121	82.31 %
Emotion	anger		28	19.05 %
	disgust		5	3.40 %
	fear		8	5.44 %
	joy		62	42.18 %
	sad		5	3.40 %
	surprise		39	26.53 %
Technology Acceptance Score	1		39	26.53 %
	2		12	8.16 %
	3		9	6.12 %
	4		34	23.13 %
	5		53	36.05 %

## 4 Experimental Design, Materials and Methods

The dataset presented in this manuscript is the first of its kind, targeting the understanding of technology acceptance for border control technologies. The dataset contains questionnaire responses collected from 147 travellers who used the border control technology at the METICOS pilot trials to capture the impact of traveller demographics, technology operational attributes, and traveller perceptions such as sentiment and emotion on overall technology acceptance. To this date, no similar dataset exists in current state-of-the-art (SOTA) studies within this scope. Therefore, we relied on previous studies to design and develop the technology acceptance questionnaire and selection of the data attributes to be collected. Additionally, the questionnaire and data attributes were carefully reviewed and confirmed by the end-users and other partners of the METICOS project consortium. This confirmation reassured us that the selected data attributes and questionnaire effectively capture travellers’ feedback on the technology acceptance of border control technologies. Initially, we conducted a literature review to identify existing approaches for technology acceptance. We found that most studies have been developed based on theoretical models. The “Technology Acceptance Model (TAM)” is the most widely used model in the field of technology acceptance due to its simplicity and strong explanatory power [1]. However, some researchers argue that TAM is overly simplistic and fails to address the complex process of technology acceptance, which relies on many variables, not just three or four. [2,3].

Hence, we did not select TAM for developing this dataset because the chosen attributes covered three distinct scopes for technology acceptance of border control technologies. The first scope relates to the effect of demographic/profile attributes on technology acceptance, such as age, gender, nationality, and education level. The second scope concerns the impact of operational and technological attributes, such as waiting time in queues, response time of technology, and queue length. The third scope involves travellers’ perception-related attributes, including sentiments, emotions, and feedback on technological aspects. These scopes were chosen based on existing state-of-the-art studies, literature reviews conducted by various partners in different work packages as part of the METICOS project, and discussions with the project's end-user partners.

Since, all the three scopes show interaction of different information attributes leading towards the acceptance of BCTs; we particularly identified informational entropy-based notion of value from the mindsponge theory, quantum mechanics, and Shannon's information theory, as strong evidence towards selecting and using all the three scopes for representing BCTs’ technology acceptance. Precisely, the theory focuses on how values are formed through the interaction of various types of information (such as demographics, operational, and perceptions information), leading to specific decision-making such as technology acceptance [4]. In our case, the first scope (i.e., effect of demographic/profile attributes of traveller), can be seen as initial filters through which individual travellers perform decision-making in terms of acceptance during interacting with BCT. For example, younger users may process technological attributes more favorably due to higher exposure and adaptability, while older individuals might assign higher entropy (uncertainty) to such systems due to limited familiarity. The second scope (i.e., impact of operational and technological attributes) interact with travellers' own values, shaped by previous experiences. For instance, travellers with previous negative experiences in long queues or high response time might assign a higher entropy to operational attributes, reducing their overall acceptance, while positive interactions decrease entropy, reinforcing into increasing overall acceptance. Finally, the third scope (i.e., travellers’ perception-related attributes, including sentiments, emotions, and feedback on technological aspects) form a dynamic layer of perception-related attributes that influence the acceptance. According to mindsponge Theory, traveller perceptions act as catalysts in the formation of values which impact directly to the decision-making interms of acceptance. Positive emotions linked to smooth travel experiences reduce entropy, enabling travellers to form value that border control technologies as beneficial. Conversely, negative feedback increases entropy, creating resistance to the overall acceptance. Hence, the combination of all the three scopes allows for a granular understanding of technology acceptance, empowering future researchers to explore the interplay between information, entropy, and values in a structured and measurable manner.

**Fig. 1:**
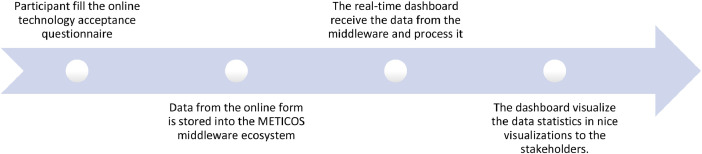
Activity flow at the METICOS pilot trials for collecting this dataset.

The method adapted to collect this dataset was based on designing an online questionnaire in a way that as soon as the travellers or participants submit their responses the data is stored securely into METICOS middleware ecosystem.^7^ The reason to collect data using an online questionnaire was due to a specific reason that we designed, developed and connected a real-time dashboard to show the dataset statistics to relevant stakeholders (i.e. border guards, consortium partners, etc.) at the METICOS pilot trials. Fig. 4 shows the snapshot of technology acceptance questionnaire used for collecting this dataset. The questionnaire consisted of three sections: 1) Profile/demographics information, 2) Operational/technological related information, and 3) Technology experience and acceptance information. Hence, covering all the three scopes discussed above. Fig. 1 depicts the overall activity flow from filling the online questionnaire from participants towards showing the dataset statistics to the relevant stakeholders via real-time dashboard at the METICOS pilot trials.

The participants for the questionnaire were recruited as a part of METICOS pilot trials. The pilot trials were executed as a part of dedicated work package (i.e. WP10: Application Trials, Evaluation and Outcomes) lead by Johanniter Österreich Ausbildung und Forschung gemeinnützige GmbH (JOAFG), Austria in the METICOS project. The pilot trials were planned and implemented at different Border Control Points (BCPs) where end-user partners of the METICOS are based, specifically in 5 countries – Estonia, Greece, Cyprus, Romania and Austria. The trials took place from January till August 2023 in PPA2, EDPMA3, CYPOL4, ITPFT5 and Austrian Border guards6 at airports in Tallinn, Athens, Larnaca, Moravita land BCP and Vienna respectively. Only adult volunteers (with a capability to consent) were recruited in each pilot trial site. The sample informed consent form used at the pilot trials will be attached as a part of this manuscript submission and is also uploaded on the data repository. The recruitment method and informed consent procedures were particularly stringent to ensure no coercion (not even soft or indirect) is exerted. The specific criteria for the selection of the volunteer participants were determined by the pilot requirements as below:

• Travellers that volunteer to participate in the research.
• Employees and other persons from the organizations of the partners that volunteer to participate in the research and members of the BCP.
• Employees of BCPs that volunteer to participate in the research.

The collected dataset from the online questionnaire was securely stored into MySQL database as a part of METICOS middleware. We used “Flask, and MySQL” libraries from Python programming language to design, implement and store the questionnaire responses into the database. In addition to this, we utilized the “Plotly” library from Python to design, develop the real-time dashboard visualizations integrated into Flask library.

## Limitations

Our dataset has several limitations. First, the dataset was collected in controlled environment with limited 147 responses during the METICOS pilot trials. Although, we were able to curate very good analysis from the collected data regarding the technology acceptance of border control technologies, but this relatively small sample size may not fully capture the diversity of travellers experiences and opinions across different demographics and geographical locations. Larger sample sizes would provide more generalizable results.. Second, the data is based on self-reported responses from travellers, which can be subject to biases such as social desirability bias, recall bias, and response bias. Participants may overestimate or underestimate their experiences and perceptions, affecting the accuracy of the data. Third, the data was collected from specific locations (four airports and one land border) within Europe. This may introduce a geographical and cultural bias, as the perceptions and acceptance levels of travellers from other regions (e.g., Asia, Africa, Americas) are not represented. Cultural attitudes towards technology and privacy can significantly influence acceptance, and this dataset may not reflect such variations. Fourth, the data collection occurred over a specific period (January to August 2023). Traveller experiences and technology acceptance can vary over time due to changes in technology, policies, and global events (e.g., COVID-19 pandemic, geopolitical tensions). The findings may not be applicable to different time frames. Finally, the data was collected in controlled environments during pilot trials, which may not fully represent real-world conditions. Factors such as stress, time pressure, and the presence of border control officers can influence travellers’ behaviour and acceptance, and these may differ in an actual border crossing scenario.

## Ethics Statement

The study protocol, including the questionnaire and data collection methods was reviewed and approved by an independent ethics committee associated with the METICOS.^8^ Written consent was obtained from each participant prior to their involvement in the METICOS pilot trial. Participants were fully informed about the purpose of the study, the nature of their involvement, and their right to withdraw at any time without any consequence. Also, participation in the METICOS pilot trials was entirely voluntary. Participants were not coerced or pressured into taking part, ensuring that their responses were freely given. Moreover, all data collected from voluntary participants were anonymized. We ensured that all the responses were treated confidentially. A sample of informed consent form is uploaded with this manuscript for your reference. The dataset is stored in a secure MySQL database as part of the METICOS middleware ecosystem. Access to the data is limited to authorized personnel only, and all data handling complies with relevant data protection regulations, including GDPR. Furthermore, the study was designed to minimize any potential risks to participants. The questions in the questionnaire were carefully reviewed to avoid any distressing or invasive topics. Finaly, participants were provided with contact information for the representative of the research team, allowing them to ask questions or raise concerns about the study at any time. Further information regarding the ethical approvals is available as public deliverables from the METICOS project at the links.^9^^,^^10^

## CRediT Author Statement

**Sarang Shaikh:** Writing – original draft, Conceptualization, Methodology, Data curation, Visualization; **Sule Yildirim Yayilgan:** Writing – review & editing, Supervision, Investigation; **Erjon Zoto:** Writing – review & editing, Supervision; **Mohamed Abomhara:** Investigation, Supervision, Ensuring data privacy & protection.

## Data Availability

Pilot) supported by ZenodoDataset of border control technologies' acceptance from travellers perspective (Original data).

## Footnotes

2 https://meticos-project.eu/esthonia/.

3 https://meticos-project.eu/greece/.

4 https://meticos-project.eu/cyprus/.

5 https://meticos-project.eu/romania/.

6 https://meticos-project.eu/austria/.

## Footnotes

7 https://meticos-project.eu/the-architecture-of-the-meticos-platform/.

8 https://meticos-project.eu/ethics-advisory-board/.

9 https://meticos-project.eu/mtc-uploads/repository/Deliverable-D1.1_H-Requirement-No.−2.pdf.

10 https://meticos-project.eu/mtc-uploads/repository/Deliverable-D1.6_EPQ-Requirement-No.9.pdf.
